# Transcranial magnetic stimulation and eating disorders, any efficacy?

**DOI:** 10.1192/j.eurpsy.2021.958

**Published:** 2021-08-13

**Authors:** J. Morais, P. Esteves, S. Fonseca

**Affiliations:** Psychiatry, Centro Hospitalar Universitário de São João, Porto, Portugal

**Keywords:** TMS, anorexia nervosa, Bulimia Nervosa, eating disorder

## Abstract

**Introduction:**

Eating Disorders (ED) tend to evolve chronically, with resistance to different therapeutic strategies. Chronicity is associated with high mortality rates, so it is necessary to study new therapeutic strategies. Transcranial Magnetic Stimulation (TMS) is a non-invasive, safe treatment method, whose application has been studied in several pathologies.

**Objectives:**

Determine the therapeutic potential of Transcranial Magnetic Stimulation in the treatment of Eating Disorders.

**Methods:**

Bibliographic review of the literature published in English in the last 10 years, in the databases Pubmed, PsycINFO and Cochrane. The keywords used were: TMS, Transcranial Magnetic Stimulation, Eating Disorder, Anorexia Nervosa, Bulimia Nervosa, Binge Eating Disorder. A review of the titles and abstracts of the resulting articles was made, and selected according to their relevance to the study.

**Results:**

Eighteen articles related to the treatment of ED with TMS were selected, either as primary or secondary outcome, of which six were review articles, ten were randomized controlled trials (RCT), one article was an oral communication and another article was a case report. Three RCTs showed improvement in bulimia nervosa, specifically in symptoms of “food craving”. Four RCT and one case report showed improvement in the symptoms of anorexia nervosa, one RCT showed no improvement in anorexia nervosa.

**Conclusions:**

TMS appears to have some therapeutic potential for the treatment of ED, particularly in reducing food craving, despite some contradictory results. This work reinforces the need for more robust studies to evaluate the effectiveness of TMS, preferably randomized, with a longer follow-up and a cost-benefit analysis.

